# Human brucellosis occurrences in inner mongolia, China: a spatio-temporal distribution and ecological niche modeling approach

**DOI:** 10.1186/s12879-015-0763-9

**Published:** 2015-02-03

**Authors:** Peng Jia, Andrew Joyner

**Affiliations:** Department of Geography and Anthropology, Louisiana State University, Baton Rouge, LA USA; Department of Geosciences, East Tennessee State University, Johnson City, TN USA

**Keywords:** Brucellosis, Geographic information systems, Remote sensing technology, Ecological niche modeling, Spatial analysis, Inner Mongolia, China, Mongolia

## Abstract

**Background:**

Brucellosis is a common zoonotic disease and remains a major burden in both human and domesticated animal populations worldwide. Few geographic studies of human Brucellosis have been conducted, especially in China. Inner Mongolia of China is considered an appropriate area for the study of human Brucellosis due to its provision of a suitable environment for animals most responsible for human Brucellosis outbreaks.

**Methods:**

The aggregated numbers of human Brucellosis cases from 1951 to 2005 at the municipality level, and the yearly numbers and incidence rates of human Brucellosis cases from 2006 to 2010 at the county level were collected. Geographic Information Systems (GIS), remote sensing (RS) and ecological niche modeling (ENM) were integrated to study the distribution of human Brucellosis cases over 1951–2010.

**Results:**

Results indicate that areas of central and eastern Inner Mongolia provide a long-term suitable environment where human Brucellosis outbreaks have occurred and can be expected to persist. Other areas of northeast China and central Mongolia also contain similar environments.

**Conclusions:**

This study is the first to combine advanced spatial statistical analysis with environmental modeling techniques when examining human Brucellosis outbreaks and will help to inform decision-making in the field of public health.

## Background

Brucellosis, a common zoonotic disease also referred to as Bang's disease, Crimean fever, Gibraltar fever, Malta fever, Maltese fever, Mediterranean fever, rock fever, and undulant fever, remains a major burden in both human and domesticated animal populations worldwide, especially in the Mediterranean region, Asia, the Middle East, Sub-Saharan Africa, Latin America and the Balkan Peninsula [1-3]. Causing more than 500,000 new human cases worldwide annually [2], Brucellosis is directly transmitted to humans via contact with animals that carry the pathogenic bacteria *Brucella* or infectious material during animal husbandry and meat processing, or indirectly through the consumption of unpasteurized dairy products [4]. Infection among different animal species is mostly attributed to different infectious agents in the genus *Brucella* (*B.*). The primary causative agents include *B. melitensis* (sheep and goats), *B. ovis* (sheep and goat), *B. abortus* (cattle), *B. suis* (swine), and *B. canis* (dog) [4-6], although more have been detected in both domesticated and wildlife species [7,8]. Brucellosis itself yields low mortality rates, but it can cause substantial disabilities and weaknesses to the human immune system [9]. The clinical presentation can be acute, subacute or chronic, varying from joint, muscle and back pain to flu-like symptoms, and even more serious conditions in different organ systems [10]. However, it most commonly targets the reproductive system, resulting in up to a 40% increase in fetal wastage during the early stages of pregnancy and up to 2% of fetal deaths during the later stages of pregnancy for expectant women [11].

Details of production, multiplication and spread of the *Brucella* spp. among livestock have been described elsewhere [12], but a brief summary is given here. Initially, *B. abortus* replicates itself in regional lymph nodes of cows. Uterine infection occurs during the second trimester and is followed by placental inflammation that develops into placental disruption and endometritis ultimately leading to fetal death, after which the fetus is normally retained 1 to 3 days in utero and delivered with numerous bacteria expelled from the genital tract and spread out by various pathways. Brucellosis is detrimental to human health, and can lead to reductions in livestock production and subsequent economic losses, reduced milk/meat production, time lost by patients from normal daily activities, and increased medical costs [13,14]. Combination therapy with long-acting oxytetracycline and streptomycin has shown to moderate the effectiveness of the disease resulting in a reduction of shedding in most cows and complete elimination of infection in some cows. However, due to low treatment success rates, many countries (e.g., United States, European Union member countries, Australia and New Zealand) adopted eradication programs to slaughter the infected cows and quarantine the exposed herdmates until they could either be slaughtered or recertified as Brucellosis-free [12,15,16].

In China, Brucellosis remains a major public health issue. Previous studies indicated that human Brucellosis was endemic in 25 of 32 provinces (or autonomous regions) of mainland China [17]. Inner Mongolia Autonomous Region has been most severely affected by Brucellosis in both humans and livestock since 1999, reporting the largest number of human Brucellosis cases across China [18,19], and accounting for approximately 40% of the total reported cases in China during 1999–2008 [18,20], 47.2% in 2010 [21], and almost 50% during 2005–2010 [19]. However, few efforts have been specifically focused on the distribution of human Brucellosis cases in Inner Mongolia. One recent study revealed potential relationships between the incidence of human Brucellosis cases in China and some environmental factors, including temperature, rainfall, hours of sunshine, relative humidity and average wind velocity [22], but additional research is needed.

The goal of this study is two-fold: 1) examine the spatio-temporal distribution of reported human Brucellosis cases and incidence rates across Inner Mongolia during 1950–2010 and 2) examine the association of environmental factors with human Brucellosis occurrences in Inner Mongolia. Geographic Information Systems (GIS), remote sensing (RS), exploratory spatial data analysis (ESDA) and ecological niche modeling (ENM) were utilized to achieve the goals of this study.

## Methods

### Ethics statement

No human subjects work was undertaken in this study. Human Brucellosis case data were extracted from annual reports and published literature. The annual reports provided summarized count data of patients reported by county and year. All data were anonymized.

### Study area

**Figure 1:**
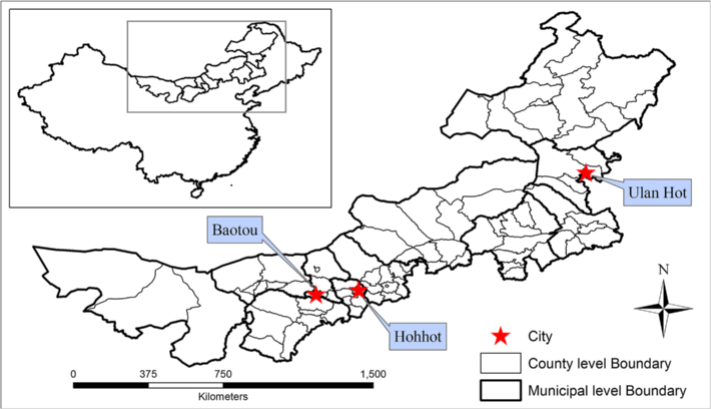
**Inner Mongolia, China.**

Inner Mongolia, located along the northern border of China, is one of five autonomous regions in which very large minority ethnic groups reside. Stretching about 2,400 km from west to east and 1,700 km from north to south, Inner Mongolia shares a long international border with Mongolia (around 3,000 km) and a shorter border with Russia (less than 1,000 km) (Figure 1). Most of Inner Mongolia contains plateau landforms averaging around 1,200 meters in altitude, covered by extensive loess and sand deposits. Inner Mongolia covers an area of 115.5 million hectares, about 12.28% of China's total land area, ranking third after Xinjiang (17.23%) and Tibet (12.75%). Approximately 67% (~78 million hectares) of the Inner Mongolian area is classified as rangeland, which consists primarily of temperate grassland [23] extending from about 40° to 50°N and from about 107° to 125°E. Due to its elongated shape, there is a wide variety of regional climates throughout Inner Mongolia, with arid and semiarid regions predominantly in the north and west, humid continental in the northeast, and subarctic in the far north bordering Russia and the Heilongjiang province of China [24].

Inner Mongolia consists of 12 administrative units at the municipal level (nine cities and three leagues), one level finer than province, and 89 subdivisions on the county level. The capital is Hohhot, and Baotou is the largest industrial city. In addition to Hohhot and Baotou, the other ten municipal units include Bayan Nur City, Chifeng City, Hulunbuir City, Ordos City, Tongliao City, Wuhai City, Wulanchabu City, Alxa League, Hinggan League, and Xilin Gol League. The earliest record related to animal Brucellosis in Inner Mongolia dates back to the year 1936 in Ulan Hot City of Hinggan League (Figure 1), called Wangye Temple at that time, where *Brucella* was found in an aborted bovine fetus.

### Data

The aggregated numbers of human Brucellosis cases in each decade from 1951 to 2000 and from 2001 to 2005 at the municipal level were extracted from published literature - the data during 1951–2003 and during 2004–2005 were originally from annual reports by the Inner Mongolia Center for Disease Control and Prevention (IMCDC) and Chinese Center for Disease Control and Prevention (CCDC), respectively [25]. The annual numbers of human Brucellosis cases along with incidence rates during 2006–2010, calculated based on total county population and reported per 100,000, were collected from annual reports by the IMCDC. Each reported case must be confirmed by both clinical signs and serological tests, or isolation in accordance with the case definition of the World Health Organization (WHO) to maintain the same case definitions as cases reported in human Brucellosis studies in China [22].

### Variables

The minimum, maximum and mean middle infrared radiation (MIR), land surface temperature (LST), and enhanced vegetation index (EVI) images were produced by Temporal Fourier Analysis [26,27], based on available 1 × 1 km data available between 2001 and 2008 from MODerate-resolution Imaging Spectroradiometer (MODIS) sensors on-board NASA’s Terra and Aqua satellites [28]. EVI is an improved version of the conventional Normalized Difference Vegetation Index (NDVI). While NDVI has been used extensively in successful studies [29], EVI functions with higher fidelity through reducing saturation of the signal at high vegetation coverage and soil background effects [30]. LST is a measure of surface, or “skin,” temperature [31]. MIR, recorded by MODIS band 7 (2,105-2,155 nm), is often associated with the water content, surface temperature, and structure of vegetation canopies [32].

The 1 × 1 km monthly values of precipitation (PREC) averaged over the 1960–1990 period were available from WorldClim (http://www.worldclim.org/), which has been widely used in a variety of species distribution and disease pattern studies [33-35]. Annual minimum, maximum and mean precipitation variables were produced by implementing raster math calculations on the 12 monthly layers available for each parameter. Additionally, altitude and 19 bioclimatic variables representative of the climatic conditions from 1950 to 2000 were analyzed for this study (Table 1). These variables, with a resolution of 1×1 km, were also available from WorldClim.

**Table 1:** **Predictor variables (data layers) in the study**

**Variable**	**Description**	**Formula**
*Remotely sensed variables*		
Evi max	Maximum EVI	EVI = 2.5 × (NIR-RED)/(NIR + 6.0 × RED–7.5 × BLUE + 1.0)
Evi mean	Mean EVI	
Evi min	Minimum EVI	
lst max	Maximum LST (°C)	
lst mean	Mean LST (°C)	split-window method
lst min	Minimum LST (°C)	
Mir max	Maximum MIR	
Mir mean	Mean MIR	MODIS band 7
Mir min	Minimum MIR	
Prec max	Maximum precipitation (mm)	
Prec mean	Mean precipitation (mm)	
Prec min	Minimum precipitation (mm)	
Alt	Altitude (m)	
*Climatic variables*		
Bio 1	Annual mean temperature (°C)	
Bio 2	Mean diurnal range(°C)	max temp - min temp
Bio 3	Isothermality	(bio2 / bio7) × 100
Bio 4	Temperature seasonality	(std. dev. / mean) × 100
Bio 5	Max temperature of warmest month (°C)	
Bio 6	Min temperature of coldest month (°C)	
Bio 7	Temperature annual range (°C)	bio5 - bio6
Bio 8	Mean temperature of wettest quarter (°C)	
Bio 9	Mean temperature of driest quarter (°C)	
Bio 10	Mean temperature of warmest quarter (°C)	
Bio 11	Mean temperature of coldest quarter (°C)	
Bio 12	Annual precipitation (mm)	
Bio 13	Precipitation of wettest month (mm)	
Bio 14	Precipitation of driest month (mm)	
Bio 15	Precipitation seasonality	(std. dev. / mean) × 100
Bio 16	Precipitation of wettest quarter (mm)	
Bio 17	Precipitation of driest quarter (mm)	
Bio 18	Precipitation of warmest quarter (mm)	
Bio 19	Precipitation of coldest quarter (mm)	
*Socioeconomic variables*		
Access	Travel time to the nearest major settlement of population size 50,000 or more (minutes)	
Pop	Population density (person/km^2^) in 2000	
Cattle05	Cattle density (heads/km^2^) in 2005	
Rumi05	Small ruminants density (heads/km^2^) in 2005	
Cattle10	Cattle density (heads/km^2^) in 2010	
Goat10	Goats density (heads /km^2^) in 2010	
Sheep10	Sheep density (heads/km^2^) in 2010	

MIR: middle infrared radiation; LST: land surface temperature; EVI: enhanced vegetation index; NIR: near infrared reflectance.

Since cattle and small ruminants have been responsible for most human Brucellosis occurrences [36], the densities of cattle and small ruminants in 2005, with a resolution of 5×5 km, were obtained from the Food and Agriculture Organization (FAO) of the United Nations and resampled to 1×1 km. In 2010, the density of cattle was available at a resolution of 1×1 km, as well as the densities of sheep and goats used for representing the density of small ruminants which was not available in 2010 [37]. The Global Rural–urban Mapping Project, Version 1 (GRUMPv1) was used to provide population numbers based on values from 1×1 km grids [38]. A global map of accessibility in 2000, available as a gridded surface at a resolution of 1×1 km with pixel values representing minutes of travel time to the nearest major settlement of population size 50,000 or more, was also utilized for this study [39]. The worldwide Land Use Systems (Version 1.1) was developed by the Land Tenure and Management Unit of the FAO and used to extract livestock-related areas.

### Spatio-temporal analysis

The annual numbers of human Brucellosis cases at the municipal level from 1950 to 2010 were temporally aggregated by decade and visually compared. The annual incidence rates of human cases from 2006 to 2010 on the county level were plotted for a visual comparison over time. The χ^2^ (chi-square) two-sample test was implemented to determine statistically significant differences in incidence rates amongst counties between each subsequent and preceding year. A natural log transformation was performed prior to each *t*-test to reduce the influence of the skewness of the distribution of incidence rate values.

To reduce the potential impact of inaccurate observed incidence rates caused by an issue of “small case numbers” or incomplete reporting, a method of spatial smoothing was applied and resulted in the replacement of raw rates with adjusted estimates based on incidence rates in neighboring counties. This can be realized by assigning either the radius (distance) of a smoothing window or by defining neighbors and number of neighbors, and is predicated on the first law of geography which states that "everything is related to everything else, but near things are more related than distant things” [40]. Using *GeoDa*, a spatial statistics and spatial data analysis software package, a first order Rook contiguity weights matrix was created for spatial smoothing, meaning that all polygons sharing a border of some length, more than a point-length border, with the target Thiessen polygon were defined as neighbors [41].

Local Moran’s I was employed to examine spatial clustering of the counties with high and low numbers of reported cases and incidence rates across Inner Mongolia, where a random permutation procedure (RPP) replicated the statistic 999 times to generate reference distributions [42]. Through comparison of reference distributions, the statistical significance of spatial clusters was tested.

### Ecological niche modeling

ENM, similar in many respects to species distribution modeling (SDM), is an innovative approach that associates the occurrence locations of infectious diseases (i.e., presence data) with a set of environmental variables resulting in a geographic range prediction for disease cases and agents. Maxent, used in this study, is a maximum entropy approach to presence-only distribution modeling that has shown a high predictive power even for small sample sizes [43,44]. However, the output from Maxent for evaluating the relative contribution of each independent variable to the model can be affected by high correlation amongst variables [45]. Prior to modeling experiments, variable correlations were examined to eliminate issues of high multicollinearity. The Maxent modeling procedure assesses the importance of the individual and collective contributions that each variable makes to the distribution of human Brucellosis cases through 1) jackknife analysis, 2) average area under the curve (AUC) values for 10 model iterations and 3) average percentage contribution of each variable to the model. The prediction was projected outside of Inner Mongolia to the country of Mongolia to the north and eight adjacent Chinese provinces that border Inner Mongolia, which from east to west include Heilongjiang, Jilin, Liaoning, Hebei, Shanxi, Shannxi, Ningxia and Gansu provinces.

Obtaining point coordinates of Brucellosis presence that best represented conditions where human Brucellosis most likely occurs proved to be challenging since original data were aggregated over administrative units. *A priori* knowledge was needed to limit and reduce the suitable habitat area. For example, it is well-known that pastoral areas have the highest incidence rates of human Brucellosis [22,46]. Instead of simply using geometric centroids of administrative units as multiple studies have done [47-51], this issue was addressed through a series of dasymetric procedures based on the verity that human Brucellosis is most likely to occur on the grasslands where both people and small ruminants (primarily sheep and goats) inhabit. The density of small ruminants in most of Inner Mongolia ranged from 0 to 58 heads/km^2^, with abnormally high densities in just a few disjunct locations. Small ruminant (sheep and goat) densities were mapped in Inner Mongolia and the densities were classified into three categories based on a quantile classification method. Goat densities greater than ~22 heads/km^2^ were classified into the highest quantile, while sheep densities greater than ~16 heads/km^2^ were classified into the highest quantile. The 16–22 heads/km^2^ range matched that of a previous study [52] that indicated that the medium density threshold for small ruminants is ~18 heads/km^2^. Thus, a threshold of 18 heads/km^2^ was considered to be a reasonable cutoff value for the medium density of small ruminants in this study. In addition to small ruminant densities, moderate elevation (800–1600 meters) is considered to be more suitable for Brucellosis occurrences [22]. Moreover, potential human Brucellosis occurrences were limited to the counties where total numbers of reported cases from 2006 to 2010 exceeded 50 persons.

Grasslands with low, moderate and high livestock densities were extracted from the land use raster layer and one centroid was created for each raster cell. The values of altitude (800–1600 meters), population density (>1 person/km^2^), small ruminant density in both 2005 and 2010 (>18 heads/km^2^) underlying each centroid were then used to further restrain the suspected occurrence locations. The case numbers from 2006 to 2010 were summed for each county and only the suspected locations falling within the counties with more than 50 cases were selected as “presence” points.

### Regression analysis

Multivariate regression models were built at the county level based on the remaining variables after removing high multicollinearity, to explore the impacts of various environmental and socioeconomic factors on the incidence rates of human Brucellosis over time. The incidence rates over counties in five individual years (2006–2010) and the average incidence rate over five years were separately used as dependent variables in six models, with the same set of independent variables used in the models and a *stepwise* method applied to each regression procedure.

## Results

### Spatial-temporal distribution of reported human Brucellosis cases

**Figure 2:**
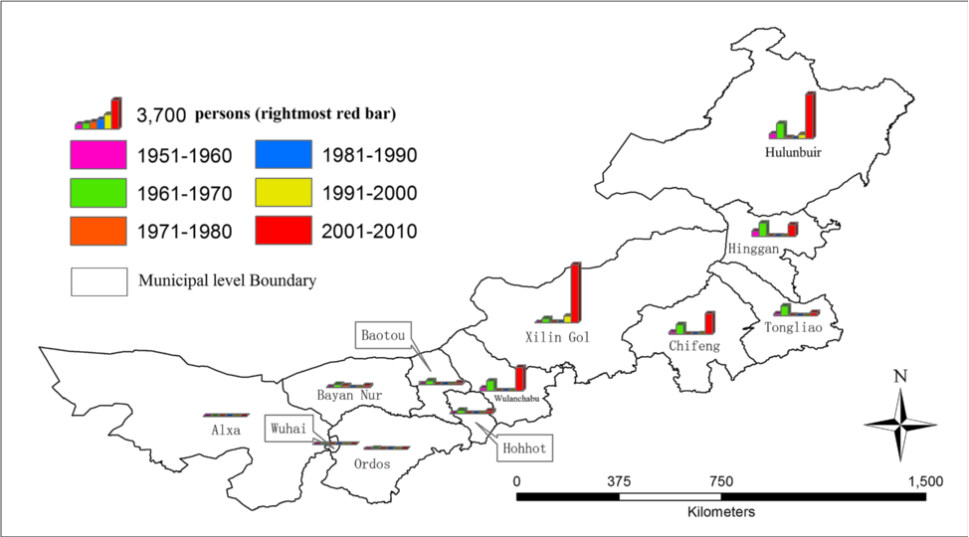
**Decadal human Brucellosis cases (persons) at the municipal level during 1951–2010.**

Figure 2 illustrates the accumulated numbers of human Brucellosis cases at the municipal level every ten years from 1951 to 2010. The 2001–2005 cases were added to the aggregated numbers from counties during 2006–2010 to generate the number of cases on the municipal level for the final decade (2001–2010). Very few occurrences were reported in the western regions when compared to the central and eastern regions of Inner Mongolia. A small historical peak of occurrences in most of the central and eastern regions occurred in the 1960s. However, prevalence subsided in the 1970s and did not resurge again until the 1990s. The increase in reported cases is extremely high for the 2001–2010 period, compared to previous decades.

**Figure 3:**
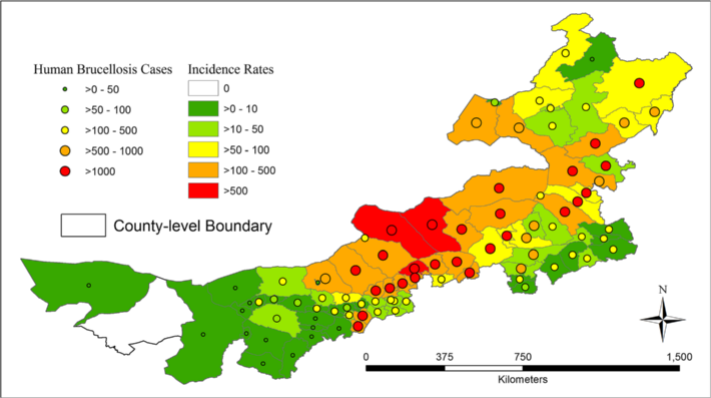
**County level spatial distribution of aggregated human cases (persons) and incidence rates (persons per 100,000 population) over 2006–2010.**

The general trend of temporal variations during the 2006–2010 period over counties showed an increase over time (Table 2), especially in most counties in the central and eastern regions with few counties remaining stable. The differences in incidence rates amongst counties between each subsequent and preceding year were statistically tested and only the increase from 2008 to 2009 was statistically significant (*p* < 0.05) (Table 3). Although non-significant increases and slight decreases were observed from 2009 to 2010, the overall increases from 2006 to 2010 were highly statistically significant (*p < 0.01*) (Table 3). Figure 3 further examined the spatial distribution of accumulated occurrences and average incidence rates over the 2006–2010 period on the county level (i.e., finer than the municipal level). Although more than 1,000 cases were reported over many counties in the central and eastern regions, the highest incidence rates (>500 persons per 100,000) were only observed in Sonid Left Banner, Abag Banner and the Bordered Yellow Banner of Xilin Gol League due to lower population totals when compared to other counties, such as Jalaid Banner of Hinggan League and Oroqen Autonomous Banner of Hulunbuir City. Compared to other cities (or leagues), the fewest human cases were reported in Bayan Nur City, Ordos City and Alxa League; no human cases have been reported in Alxa Right Banner of Alxa League through 2006–2010.

**Table 2:** **Descriptive statistics of raw and natural log-transformed incidence rates of Brucellosis occurrences in humans by county during 2006-2010**

**Year**	**Counties**	**Raw mean**	**Log mean**	**Log Std. deviation**	**Log Std. error mean**
2006	89	76.149	1.065	4.721	0.500
2007	89	80.963	1.224	4.600	0.488
2008	89	110.497	1.946	4.251	0.451
2009	89	158.035	3.135	3.059	0.324
2010	89	128.852	3.313	2.894	0.307

**Table 3:** **Results (equal variances assumed) of** ***t*** **-test for the difference of incidence rates of human Brucellosis occurrences between each subsequent and preceding year during 2006-2010**

	**Levene’s test**		***t*** **-test for equality of means**						
**Year**	**F.**	**Sig.**	**t**	**df**	**Sig.(2-tailed)**	**Mean difference**	**Std. error difference**	**95% lower**	**95% upper**
2007 v.s. 2006	0.113	0.737	−0.228	176	0.820	−0.159	0.699	−1.538	1.220
2008 v.s. 2007	0.851	0.358	−1.088	176	0.278	−0.722	0.664	−2.032	0.588
2009 v.s. 2008	5.090	0.025	−2.142	176	0.034	−1.189	0.555	−2.285	−0.094
2010 v.s. 2009	0.399	0.528	−0.398	176	0.691	−0.178	0.446	−1.058	0.703
2010 v.s. 2006	17.594	0.000	−3.830	176	0.000	−2.248	0.587	−3.406	−1.090

**Figure 4:**
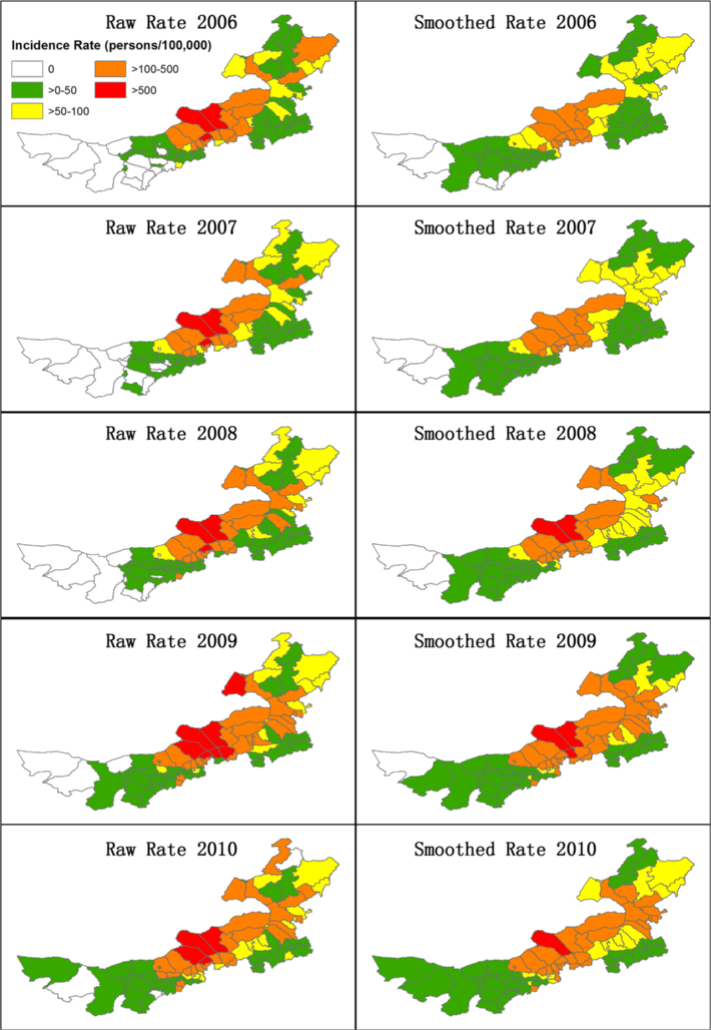
**Raw and spatially smoothed incidence rates of human Brucellosis cases.**

**Figure 5:**
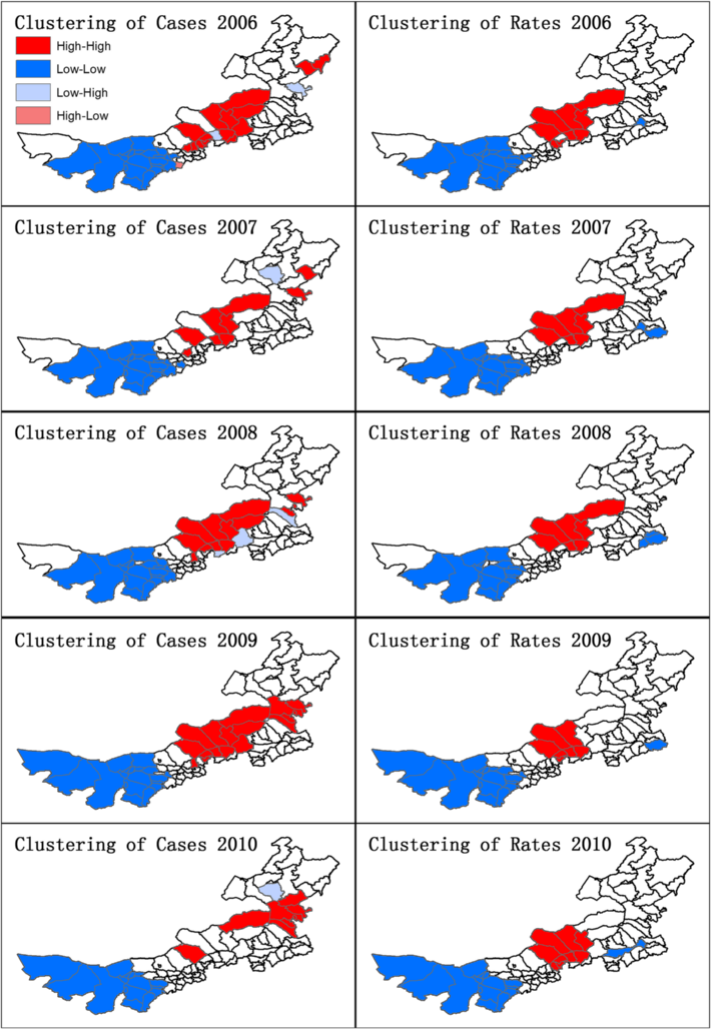
**Spatial clustering of human Brucellosis cases and incidence rates.**

The raw and spatially-adjusted incidence rates were mapped in the left and right column within Figure 4. In the years of 2006 and 2007, the high rates in Sonid Left Banner, Abag Banner and Bordered Yellow Banner of Xilin Gol League were smoothed by their neighbors. However, the high rates in Sonid Left Banner and Abag Banner persisted in both raw and adjusted calculations since 2008. After decreases in 2010, only the high rate in Sonid Left Banner remained. The higher rates in central Inner Mongolia counties have been confirmed by the clustering trend in Figure 5, where Xilinhot City, Abag Banner, Sonid Left Banner, Sonid Right Banner, Plain and Bordered White Banner, Xulun Hoh Banner formed a high cluster throughout 2006–2010 (except Xilinhot City in 2009). A major low cluster was formed by Bayan Nur City, Ordos City and Alxa League, and a minor low cluster was observed within and adjacent to Horqin Left Back Banner of Tongliao City.

### Ecological niche modeling

Two ecological niche models (ENM_1 and ENM_2) were produced to predict the potential risk areas for human Brucellosis. The ENM_1 used all suspected occurrence points available based on five years of reported human cases (2006–2010) and 17 environmental and socioeconomic variables after removing some variables that were found to have high multicollinearity with other variables. Of the total suspected points, 25% were randomly excluded from modeling and used for model testing and validation. The ENM_2 used the same 17 variables as ENM_1, and the suspected occurrence points produced based on three years of reported human cases (2006–2008), with the last two years of data (2009–2010) withheld from the model to be used as the testing (validation) dataset.

**Figure 6:**
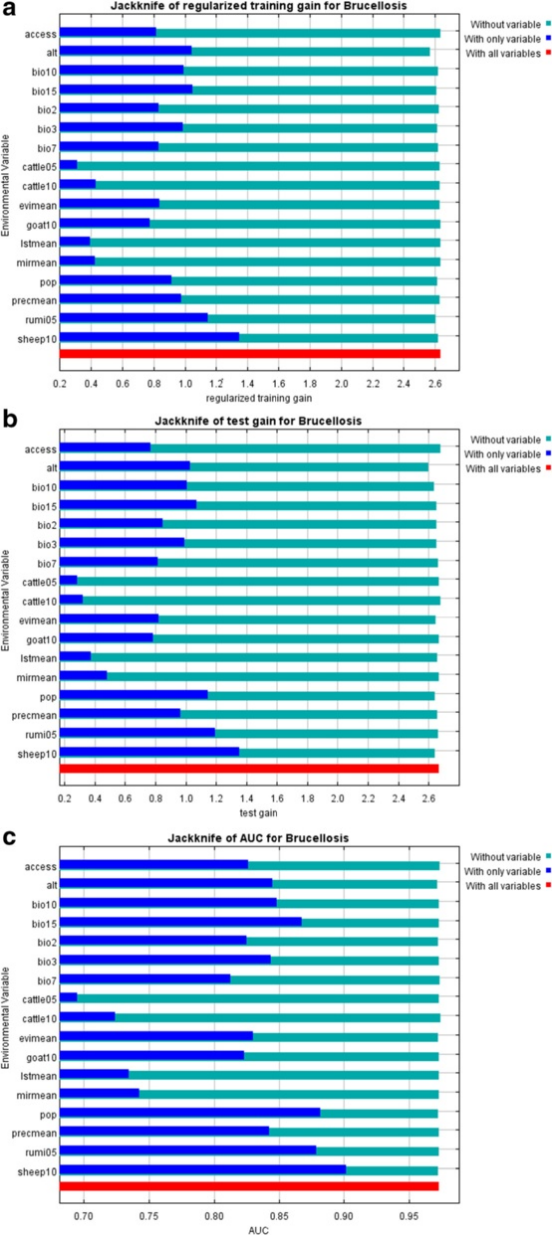
**Jackknife analysis results. (a)** The Jackknife of regularized training gain test which predictor (variable) contributes most to the model **(b)** The Jackknife of test gain uses the test samples (not used in the model) to measure how each predictor (variable) contributes to the average likelihood of presence locality **(c)** The Jackknife of AUC assesses differences in significance to show that the model performs better than random based on each variable.

Table 4 showed the relative contributions of 17 variables to two models. The three most important variables in ENM_1 were the density of sheep in 2010, density of small ruminants in 2005 and altitude, followed by three environmental variables, precipitation seasonality (bio 15), temperature annual range (bio 7) and isothermality (bio 3). There were no significant differences in the relative contribution of each variable to ENM_2. The first two most important variables were still the densities of ruminants in 2005 and sheep in 2010, followed by altitude and the same three environmental variables in a slightly different order. Accessibility also provided some explanatory capability in both models. The ENM_1 had high training (0.976) and test AUCs (0.973), indicating a good predictive fit for both the training and test sets (Table 4). The test set in the ENM_2 was more independent from the training set than in the ENM_1, so the test AUC was slightly lower but still high (0.936). The jackknife analysis results revealed the individual influences of each variable, which was consistent with the relative contributions (Figure 6, for ENM_1). The model was minimally weakened when any variable was omitted with all other variables remaining because of their relative influence.

**Table 4:** **Analysis of variable contributions and model assessment**

	**ENM**_**1**		**ENM**_**2**	
**Variable**	**Percent contribution**	**Permutation importance**	**Percent contribution**	**Permutation importance**
Sheep10	23.4	10.5	22	8.1
Rumi05	20.2	9.8	22.6	4.8
Alt	15.3	25.2	13.8	32.9
Bio 15	14.7	8.7	11.8	6.8
Bio 7	9.5	3.1	12.1	9.5
Bio 3	7.3	6.0	6.2	1.9
Access	5.9	1.4	6.1	0.7
Bio 10	1.3	6.6	2.4	5.9
Pop	0.9	12.5	1.0	9.6
Bio 2	0.7	3.4	0.4	4.1
Cattle05	0.5	2.3	0	1.0
Evi mean	0.2	2.8	0.2	3.6
Cattle10	0.2	1.2	0.2	1.3
Prec mean	0.1	5.3	0.5	6.8
lst mean	0.1	0.6	0.1	1.8
Goat10	0	0.3	0.5	0.8
Mir mean	0	0.5	0	0.2
	ENM_1		ENM_2	
Training gain	2.634		2.614	
Test gain	2.667		1.252	
Training AUC	0.976		0.975	
Test AUC	0.973		0.936

**Figure 7:**
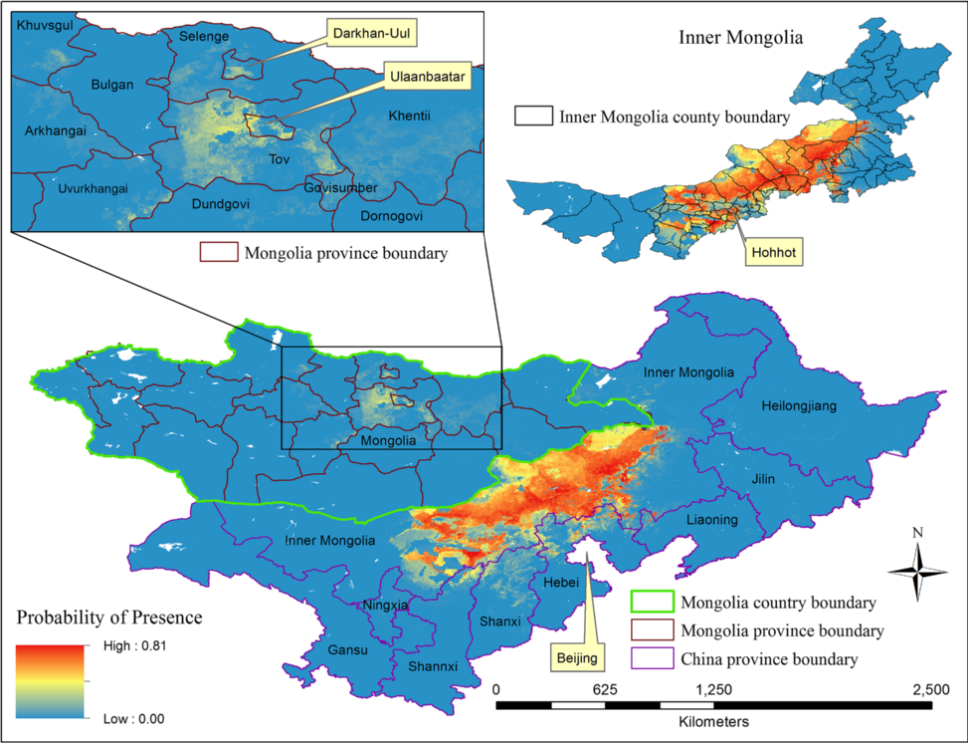
**Probability of presence of human Brucellosis in nine Chinese provinces and the country of Mongolia.**

A map of the probability of presence of human Brucellosis from ENM_1, covering Inner Mongolia, eight adjacent provinces and the country of Mongolia, was shown in Figure 7. The red and blue areas indicated high and low probability of presence of human Brucellosis, respectively, based on the similarity of environmental and socioeconomic conditions in areas of known human Brucellosis occurrences. Most regions in central Mongolia and some in eastern Mongolia were shown with high probabilities of presence of human Brucellosis. The probabilities were averaged over counties in Inner Mongolia based on counties that were classified into different levels of risk (Table 5). Human Brucellosis occurrences may be present outside of Inner Mongolia, especially in northern Liaoning, Hebei, Shanxi and Shannxi provinces. Some regions in the country of Mongolia had similar environmental and socioeconomic conditions to the suspected occurrence locations in Inner Mongolia including Ulaanbaatar, Darkhan-Uul, Tov, Selenge, Khuvsgul, Khentii, Govisumber, Dundgovi, Uvurkhangai, and Arkhangai provinces.

**Table 5:** **Counties (cities, districts or banners) with different extents of probability for human Brucellosis**

**Probability of presence**	**Counties (ordered by probability from high to low)**
>0.5 – 0.57	West Ujimqin Banner, Plain and Bordered White Banner, Duolun County, Xulun Hoh Banner
>0.4 – 0.5	Bordered Yellow Banner, Xilinhot City, Sonid Right Banner, Abag Banner, Darhan Muminggan United Banner, Tumed Left Banner, Siziwang Banner, Hohhot City, Tumed Right Banner, East Ujimqin Banner, Hexigten Banner, Jungar Banner, Baiyun Mining District
>0.3 – 0.4	Urat Middle Banner, Horinger County, Linxi County, Chahar Right Back Banner, Sonid Left Banner, Urat Front Banner, Guyang County, Shangdu County, Dalad Banner, Baotou City
>0.2 – 0.3	Togtoh County, Qingshuihe County, Huade County, Chahar Right Middle Banner, Harqin Banner, Baarin Right Banner, Xinghe County, Donghe District, Wuchuan County, Hanggin Banner
>0.1 – 0.2	Liangcheng County, Zhuozi County, Taibus Banner, Otog Banner, Chifeng City, Chahar Right Front Banner, Ongniud Banner, Wuyuan County, Baarin Left Banner, Ejin Horo Banner, Dengkou County, Linhe District
>0.01 – 0.1	Hanggin Rear Banner, Uxin Banner, Horqin Right Front Banner, Jining District, Fengzhen City, Jarud Banner, Horqin Right Middle Banner, Ningcheng County, Aohan Banner, Erenhot City, Holingol City, Ar Khorchin Banner, Otog Front Banner, Tuquan County, New Barag Left Banner, Wuhai City, New Barag Right Banner, Urat Rear Banner, Ulan Hot City, Evenk Autonomous Banner

### Regression analysis

Each of the 17 remaining layers were used in the county-level linear regression models with six variables describing different density types, from which six corresponding variables of total county numbers were derived and also used in regression models (Table 6). The results from multivariate regression were shown in Table 7. The means of LST and MIR provided explanatory capabilities to the models in all years but 2007, where another temperature variable named isothermality (bio 3), calculated by dividing mean diurnal range (bio 2) by temperature annual range (bio 7), substituted for the means of LST and contributed to the model in the same direction. The counties with lower average temperature tended to have higher reported incidence rates of human Brucellosis, while the counties with higher average MIR tended to have higher incidence rates. The total numbers of sheep in 2010 had consistently positive impacts on the occurrences of human Brucellosis in all years. Altitude and total number of cattle in 2005 had positive impacts on the incidence rates, and accessibility had opposite impacts on the incidence rates. However, the impacts by those three variables were only observed in one year. The densities of small ruminants in 2005 and cattle in 2010, isothermality, and accessibility were also influential in the ENM models.

**Table 6:** **Variables of total numbers (persons or heads) derived from original variables of densities (persons/km** ^**2**^ **or heads/km** ^**2**^ **)**

**Original layer**	**New name**	**Derived variable**	**Description**
Pop	pop den	pop tot	Total number of population in 2005
Cattle05	ca05 den	ca05 tot	Total number of cattle in 2005
Rumi05	ru05 den	ru05 tot	Total number of small ruminants in 2005
Cattle10	ca10 den	ca10 tot	Total number of cattle in 2010
Goat10	go10 den	go10 tot	Total number of goats in 2010
Sheep10	sh10 den	sh10 tot	Total number of sheep in 2010

**Table 7:** **Significant independent variables for the incidence rates of human Brucellosis cases in different years (** ***p*** **< 0.05)**

	**2006**	**2007**	**2008**	**2009**	**2010**	**Average**
*Variables*						
Bio 3	−0.436	-	-	-	-	-
lst mean	-	−0.766	−0.767	−0.730	−0.965	−0.990
Mir mean	0.227	0.752	0.748	0.742	0.877	0.905
Access	-	-	-	-	−0.235	−0.220
Alt	0.339	-	-	-	-	-
ca05 tot	0.246	-	-	-	-	-
sh10 tot	0.194	0.290	0.314	0.302	0.366	0.305
*Assessment*						
R^2^	0.317	0.357	0.377	0.356	0.466	0.425
Adjusted R^2^	0.275	0.334	0.355	0.333	0.441	0.397

## Discussions

Multiple studies have explored the spatial patterns of human Brucellosis in various countries including Azerbaijan [4], Italy [52], Germany [53], United States [54], Ecuador [36], and mainland China [14,19,55]. However, the most suitable study area for Brucellosis in China, Inner Mongolia, has not been well-studied. Moreover, limited efforts have primarily focused on the environmental dynamics of human Brucellosis occurrences [22]. To fill the gaps, this study systematically examined the spatial-temporal distribution of reported human Brucellosis cases and incidence rates at the municipal level from 1950 to 2010 and at the county level from 2006 to 2010, as well as the impacts of environmental and socioeconomic factors on human Brucellosis occurrences at point-level and county-level locations.

The study represents the first effort to combine GIS, RS and ENM techniques for the study of human Brucellosis. With the rapid spread of advanced spatial techniques and fast growing volume of high-resolution spatio-temporal data, GIS and RS are playing an increasingly vital role in medical geography and spatial epidemiology over recent years. GIS and RS provide a powerful toolset and diversity of data sources for ENM; ENM in turn has advanced conventional spatio-temporal analysis by adding more mathematical and computational complexity.

The probability of presence for human Brucellosis based on the ENM represents the extent of the similarity of environmental and socioeconomic conditions at each location to those most suitable for human Brucellosis. The locations with high predicted probability of presence do not necessarily have a large number of reported cases in the past. They may just have suitable conditions and, consequently, the potential for future occurrences. Pilot surveillance programs should be launched to determine if any under reporting or reporting biases exists at those locations.

From the perspective of historical variations, although there has been a surprising increase from 1950 to 2010, increasing accessibility to health facilities for diagnosis and reporting may be partly responsible for the perceived increase in human Brucellosis cases, especially in Brucellosis epidemic regions where the incidence rates of human Brucellosis cases increased dramatically between 2001 and 2010 (e.g., Hulunbuir and Xinli Gol). The number of health facilities has also dramatically increased and improved in Inner Mongolia since 2003 when reporting networks were established. Surveillance is typically based on the diagnosis of human cases in the nearest health facilities, which are then communicated to the IMCDC by municipal health departments and subsequently forwarded to the CCDC through the National Notifiable Disease Surveillance System. This procedure may be affected by a lack of accurate diagnosis, imprecise symptoms, uneven case detection efforts, and reporting bias; therefore a spatially smoothed approach was used for reducing spurious outliers. Reporting bias may also, to some extent, result in a mismatch between highly-suspected occurrence points and areas with high risk for Brucellosis predicted by ENM models.

Inner Mongolia is well-known for its extensive prairies and grass-fed livestock including cattle and sheep, which are often the carriers for the bacteria *Brucella*. The region contained approximately 18.2% of the total sheep population during 2005–2010, larger than any other province in mainland China [56]. Consequently, Inner Mongolia is considered a focal area for the study of human Brucellosis. A descriptive study in Inner Mongolia revealed that occupation (agriculture worker, shepherd, butcher, slaughter-house worker, and cattle dealer), risky practices (handling of ruminant abortions, skinning of stillborn lambs and kids, and crushing the umbilical cord of newborn lambs and kids with teeth), and certain dietary preferences (consuming unpasteurized and unboiled milk and fresh cheese) were correlated with the occurrence of Brucellosis in humans [18]. Larger numbers of reported human cases during 1999–2008 occurred predominantly in a specific gender (male, 70.2%) and age range (30–59 years old, 64.7%) and is likely a result of these groups participating in risky occupations and practices. Occupational features of Brucellosis also help to explain why more cases are reported in the east (humid, grassland-dominant) than west (arid and semiarid, desert-dominant) and in rural areas over urban areas. Additional educational programs should be launched, specifically aimed at enhancing the awareness of work-place Brucellosis prevention for certain groups.

Brucellosis is most often carried by domesticated animals and evidence from a model of animal-human Brucellosis transmission shows that 90% of human Brucellosis cases are small-ruminant derived in Mongolia [6]. In China, higher incidence rates of human Brucellosis were positively associated with the density of sheep and goats [22] and approximately 84.5% of the total human cases were found to be infected by *B. melitensis* [17]. Especially in Inner Mongolia, this proportion reached 90.25% or higher during 1996–2010 [21]. Therefore, small ruminant density in 2005 is used as an indicator for the possibility of human occurrences, and sheep and goats are combined to represent small ruminant densities in 2010 when small ruminant density is not available. Cattle was not associated with human Brucellosis occurrences in a previous study in China [22]. In addition to livestock density, nomadicity might play an important role in the transmission of Brucellosis among animals in Inner Mongolia through the mixing of herds, which can affect the transmission in humans. This is a limitation of this study due to the complex movement of livestock.

The vaccination rate for sheep in Inner Mongolia was previously only 31.6% [25]. A study concluded that vaccinating adult sheep alone is insufficient in eradicating Brucellosis based on the prediction that Brucellosis would persist for a long period of time even though all sheep were supposedly vaccinated twice per year [21]. Since 2011, the government in Inner Mongolia began to require vaccinations for all sheep twice per year. Given that infected sheep are still common across Inner Mongolia, especially amongst local herdsman, simultaneous disinfection and vaccination was suggested, as well as regular sheep surveillance which has been realized for some areas in China [21].

Environmental modeling for human Brucellosis is uncommon because Brucellosis is not as climate-sensitive as some infectious diseases, such as malaria [57,58] and dengue fever [45,59] among other diseases. However, varying environmental conditions can result in a lack of water or grass in the pasturing areas, which increases disease susceptibility in animals with low resistance. Altitude especially can play an important role in the types of vegetation that can grow in certain areas, precipitation levels, temperature ranges, and other environmental conditions important to the survival and transmission of *Brucella*. Consequently, altitude was the most influential abiotic variable within the ENM. Additionally, Brucellosis is capable of being transmitted by fomites. The *Brucella* organism can survive for several months in water, aborted fetuses, manure, hay, contaminated equipment and clothes, and especially in conditions with high humidity, low temperature, and no sunlight [5]. Although the theory is on a micro-level, similar environmental impacts have been revealed by a macro-level study, where lower temperature and less sunshine in winter and spring, and a 1–2 month incubation period have been associated with epidemic peaks from March to August, especially in the month of May [22]. Inhabitants of Inner Mongolia also spend more time working outside during epidemic months than during the harsh winter months, increasing their exposure to Brucellosis infection. In addition to environmental and biological determinants of the organism, different cultural and livestock management practices among communities in the highlands might impact differences in risk factors, but these differences warrant additional epidemiological field work before they can be confirmed.

After Inner Mongolia, the five provinces with the most human Brucellosis cases reported during 2005–2010 (Shannxi, Heilongjiang, Hebei, Jilin and Shanxi) all share a border with Inner Mongolia, which was also detected as the primary cluster of incidence rates [55] and accounted for most major occurrences of human Brucellosis cases in China [19]. Therefore, the ENM prediction was extended outside of Inner Mongolia to all bordering counties. Some human cases were previously reported in non-epidemic parts of China, such as Guangdong and Fujian provinces in southeastern China, which might be attributed to increased transportation of un-quarantined and unvaccinated animals and travelling of people from epidemic areas [11,18,22]. It is recommended to examine travel history as part of a more inclusive epidemiological investigation in non-endemic regions.

A major limitation of the study pertains to the quality of disease data that were available (as is the case with most ENM and SDM research [47-51]) because the true occurrence locations were not available. Brucellosis is not a contagious disease among humans, so predicting the high risk areas of human Brucellosis requires knowledge of the locations where people are most likely infected. The dasymetric procedure provided a much more certain location-to-environment relationship for modeling purposes and was superior to the alternative use of average environmental conditions at the county or municipal levels. According to the study of the occupation of patients during the 1991–2005 period in Inner Mongolia, peasants and herders consisted of 79.36% of all cases [25]. This provided further evidence in selecting appropriate locations for human Brucellosis. Other limitations include the inability to consider the differences in the accessibility to health facilities and diagnostic services between the counties reporting larger and smaller numbers of human cases.

This study provides a new direction for human Brucellosis research and greatly contributes to our knowledge of the roles that various environmental and socioeconomic factors play in the distribution and spread of human Brucellosis occurrences. Moreover, the output of this study will promote comparisons with future research at an international scale and provide a new perspective to inform decision-making in the field of public health in Inner Mongolia. This study also serves as a predecessor to broader and more in-depth studies of human Brucellosis in Inner Mongolia. Expansions in both spatial and temporal scales are warranted and could provide further insight.

## Conclusions

The main conclusions of this study are as follows: 1) Human Brucellosis occurrences exhibit a spatial trend gradually changing in the number of cases from east and central Inner Mongolia to the west, with the incidence rates in central Inner Mongolia higher than those of other regions; 2) The major variables contributing to the model include the density of small ruminants (especially sheep), altitude, precipitation seasonality, and temperature annual range; 3) The incidence rates in the highest regions may be skewed by smaller populations; 4) The density of sheep (positively) and the mean of LST (negatively) and MIR (positively) were correlated with the incidence rates of human Brucellosis at the county level; 5) It is expected that parts of Mongolia and northern Hebei, Shanxi and Shannxi, in addition to Inner Mongolia, have some risk for human Brucellosis occurrence, based on their suitable conditions; 6) Counties in Inner Mongolia are classified into a series of categories with differing levels of probability for the presence of human Brucellosis.
